# Insight into Recent Advances in Degrading Androgen Receptor for Castration-Resistant Prostate Cancer

**DOI:** 10.3390/cancers16030663

**Published:** 2024-02-04

**Authors:** Qiao-Hong Chen, Erick Munoz, Dennis Ashong

**Affiliations:** Department of Chemistry and Biochemistry, California State University, Fresno, CA 93740, USA; eim187@mail.fresnostate.edu (E.M.); dashong@mail.fresnostate.edu (D.A.)

**Keywords:** androgen receptor, castration-resistant prostate cancer, protein degradation, PROTACs, hydrophobic tag, molecular glue, ubiquitin proteosome system, autophagy

## Abstract

**Simple Summary:**

A promising approach to combat aggressive prostate cancer involves androgen receptor degraders, which break down the crucial protein driving cancer progression. In contrast to traditional drugs, these degraders offer a potential solution to drug resistance. Methods like PROTACs have shown promise in reducing androgen receptor levels in cells and clinical benefits in patients. Six PROTACs and two other degraders have entered early clinical trials for potential treatment. While progress has been made in understanding these degraders, it is essential to stay updated on emerging developments. This article provides an overview of advancements in this field since 2020.

**Abstract:**

Induced protein degradation has emerged as an innovative drug discovery approach, complementary to the classical method of suppressing protein function. The androgen receptor signaling pathway has been identified as the primary driving force in the development and progression of lethal castration-resistant prostate cancer. Since androgen receptor degraders function differently from androgen receptor antagonists, they hold the promise to overcome the drug resistance challenges faced by current therapeutics. Proteolysis-targeting chimeras (PROTACs), monomeric degraders, hydrophobic tagging, molecular glues, and autophagic degradation have demonstrated their capability in downregulating intracellular androgen receptor concentrations. The potential of these androgen receptor degraders to treat castration-resistant prostate cancer is substantiated by the advancement of six PROTACs and two monomeric androgen receptor degraders into phase I or II clinical trials. Although the chemical structures, in vitro and in vivo data, and degradation mechanisms of androgen receptor degraders have been reviewed, it is crucial to stay updated on recent advances in this field as novel androgen receptor degraders and new strategies continue to emerge. This review thus provides insight into recent advancements in this paradigm, offering an overview of the progress made since 2020.

## 1. Introduction

### 1.1. Androgen Receptor and Prostate Cancer

The androgen receptor (AR), NR3C4 (nuclear receptor subfamily 3, group C, gene 4), is an androgen-dependent transcription factor with four functional domains: the ligand binding domain, DNA binding domain, hinge region, and *N*-terminal domain. Two endogenous androgens, testosterone, and its more potent metabolite, 5*α*-dihydrotestosterone, bind to the ligand binding domain and initiate the transcriptional pathway. This transcriptional signal pathway has been established as the primary driving force behind prostate cancer cell growth and metathesis [1]. Specifically, heat shock proteins, as chaperone proteins, stabilize the AR in the cytoplasm. The binding of 5*α*-dihydrotestosterone to the ligand binding pocket of the AR alters its conformation, causing the dissociation of chaperone proteins from the AR and exposing the AR to the nuclear localization signal. This process induces the interaction between the *N* and C termini of the AR, enabling the binding of AR dimers to importin-α for translocation into the nucleus [2]. Upon entering the nucleus, AR dimers bind to androgen response elements located in the promoter regions of target genes, such as prostate-specific antigen and transmembrane protease serine 2. This binding promotes transcription, leading to responses such as cell proliferation and survival. The search for the first generation of AR antagonists began in the late 1960s and early 1970s, leading to the FDA approval of three first-generation AR antagonists: flutamide, nilutamide, and bicalutamide for prostate cancer treatment. This validated the concept that blocking the function of the AR could suppress prostate cancer cell growth [1]. The AR signaling pathway remains a pivotal drug target for the treatment of both hormone-sensitive and castration-resistant prostate cancer. This is further affirmed by the FDA’s recent approval of three second-generation AR antagonists for patients at different stages of prostate cancer since 2012 [3,4,5].

The benefits and limitations of AR antagonists as therapeutic strategies for prostate cancer were overviewed in our earlier review article [6]. The intrinsic and acquired resistance to AR antagonists posed the main concern of this group of therapeutics. One of the most established mechanisms behind the resistance to the current FDA-approved second-generation AR antagonists is the reactivation of AR signaling, driven by factors documented in the literature [7,8,9,10,11,12,13]. These factors include:

- AR overexpression.
- AR gene amplification.
- Point mutations in the ligand-binding domain of the AR, creating a larger androgen binding pocket and converting AR antagonists to agonists.
- AR splice variants (e.g., AR-V7) lacking the ligand-binding domain. These variants are constitutively active and activate downstream transcription independently of ligands. This explains the limited efficacy of current FDA-approved AR antagonists for castration-resistant prostate cancer patients with AR splice variances [1,14,15,16].
- The hypersensitive pathway [17].
- The promiscuous pathway [17].
- Coactivators and corepressors [17].
- The outlaw pathway [17].

Some non-AR pathways, such as the AR bypass mechanism [18] and androgen-independent lurker cells [19], also contribute to the therapy resistance of prostate cancer.

### 1.2. Targeting Proteins for Degradation

Targeting proteins for degradation emerges as a novel and innovative therapeutic modality, aiming to completely remove target proteins from inside and outside cells rather than merely suppressing their functions [20]. The first application of induced protein degradation in drug discovery dates back to the development of heat shock protein 90 (HSP90) inhibitors as anticancer agents in 1990. The reported HSP inhibitors have been revealed to bind to the ATP-binding domain, altering the HSP90 chaperone cyclin and resulting in the degradation of HSP90 client proteins. This therapeutic approach gained momentum with the advent of proteolysis-targeting chimeras (PROTACs) and molecular glues, later receiving an endorsement from the macroautophagic and endolysosomal protein degradation pathways [20]. Both PROTACs and molecular glues hijack the ubiquitin-proteasome system, an indispensable pathway for the degradation of damaged or misfolded proteins.

### 1.3. Targeting AR for Degradation

Targeting AR for degradation emerges as a complementary approach to AR antagonists, offering potential benefits outlined below:

- It degrades the AR rather than antagonizes its function, overcoming resistance caused by the reactivation of AR signaling through factors discussed in Section 1.1. This is evidenced by the superior performance of the enzalutamide-based PROTAC (ARCC-4) over enzalutamide in prostate tumor cells with point mutations (F876L and T877A) in the AR [21].
- It is “event-driven” rather than “occupancy-driven” in AR antagonists, potentially enhancing therapeutic potency while reducing systemic drug exposure.
- It can be synergistic with AR antagonists.

The chemical structures, structure–activity relationships, and in vivo and in vitro data of AR degraders have been extensively reviewed in the literature [22,23,24]. This article provides an overview of strategies for degrading the AR developed since 2020, highlighting the current trends in this rapidly growing research field. Section 2 includes information on AR degradation under physiological conditions, offering comparisons and insights into diverse induced AR degradation. Notably, the classification systems for AR degraders have been inconsistent and confusing, particularly for monomeric degraders. In this review, AR degraders are classified below based on degradation mechanisms:

- Bifunctional AR PROTACs, which hijack the ubiquitin–proteasome system (Section 3).
- Hydrophobic tagged chimeric degraders, which simulate misfolded AR (Section 4).
- Monomeric AR degraders, which destabilize the AR via diverse mechanisms (Section 5).
- Molecular glues (Section 6).
- Autophagic degradation of AR (Section 7).

## 2. AR Degradation Pathways under the Physiological Conditions

**Figure 1:**
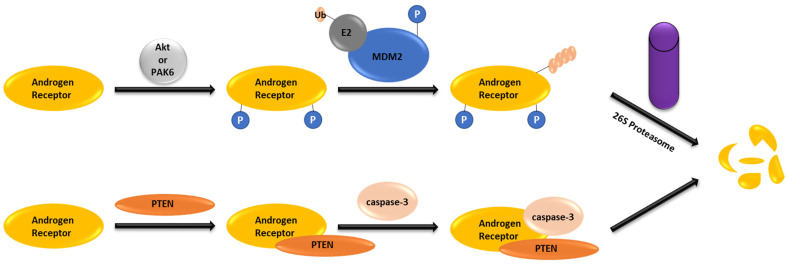
AR degradation pathways under physiological conditions. Notes: Phosphorylation of both the AR and MDM2 for increased association. PTEN exposes the active site on the AR to caspase-3.

As illustrated in Figure 1, the AR undergoes degradation either via the ubiquitin–proteasome system or via PTEN-caspase-3 to maintain appropriate levels under physiological conditions [25]. The ubiquitin–proteasome pathway plays a crucial role in maintaining protein and cellular homeostasis by covalently attaching ubiquitin molecules to a target protein, facilitating its degradation by 26S proteasome. This process involves the activation of ubiquitin by the E1 ubiquitin-activating enzyme. The activated ubiquitin is then transferred over to the ubiquitin-conjugating enzyme E2. From the E2 enzyme, ubiquitin is transferred over to an E3 ligase, which is then transferred over to the target protein. The protein tagged with ubiquitin is recognized and degraded by 26*S* proteasome. About 600 enzymes belong to the E3 ligase family, but the most established E3 ligases include Skp1-Cullin-F box (SCF), inhibitor of apoptosis protein (IAP), mouse double minute 2 homolog (MDM2), cereblon (CRBN), and von Hippel–Lindau (VHL) [26]. MDM2 E3 ligase is involved in systematic AR degradation by the ubiquitin-proteasome pathway. Phosphorylation of AR at Ser^213^ and Ser^791^ through the phosphatidylinositol-3-hydroxy kinase (PI3K)/Akt pathway leads to the destruction sequence for recognition by MDM2 E3 ligase. The AR PEST sequence is in the hinge domain, which is enriched in proline, glutamic acid, serine, and threonine and serves as the UPS degradation motif [27,28]. MDM2 forms a complex with the AR by interacting with its *N*-terminal domain and DNA binding domain, leading to AR ubiquitination and subsequent degradation mediated by 26S proteasome [29].

Alternatively, independent of the 26S proteasome, AR degradation can be facilitated by the tumor suppressor PTEN (phosphatase and tensin homolog deleted from chromosome 10) through the caspase-3-dependent pathway (Figure 1). The interaction between the AR DNA-binding domain and the PTEN phosphatase domain exposes the AR active site for recognition by caspase-3. PTEN-induced caspase-3 activation leads to the cleavage and degradation of the AR into three fragments in the cytoplasm [25,30].

## 3. AR PROTACs

The concept of proteolysis-targeting chimera (PROTAC) was initially documented by Professor Deshaires at the California Institute of Technology and Professor Crews at Yale University in 2001 in their joint paper in the *Proceedings of the National Academy of Sciences* [31]. Since then, PROTACs have emerged as an innovative strategy for selectively degrading target proteins, representing the most defined approach to induced protein degradation. Various preclinical and clinical data have validated PROTAC’s capability to combat resistance mechanisms. In contrast to occupancy-driven pharmacology, PROTACs utilize an event-driven mode of action, down-regulating protein levels by hijacking the ubiquitin–proteasome system. Generally, a PROTAC is a heterobifunctional molecule consisting of three parts: a targeted protein ligand, an E3 ubiquitin ligase ligand, and an appropriate linker connecting the two. A PROTAC molecule forms a ternary complex with the target protein and an E3 ligase, creating proximity between the target protein and the ubiquitin–proteasome system, ultimately degrading the target protein [32]. One key aspect of PROTACs is that the binding site of the target protein does not need to be functional. Additionally, in contrast to small molecule inhibitors and antagonists, temporary binding is sufficient for PROTACs to induce degradation due to the low correlation between binding affinity and degradation capability [33].

**Figure 2:**
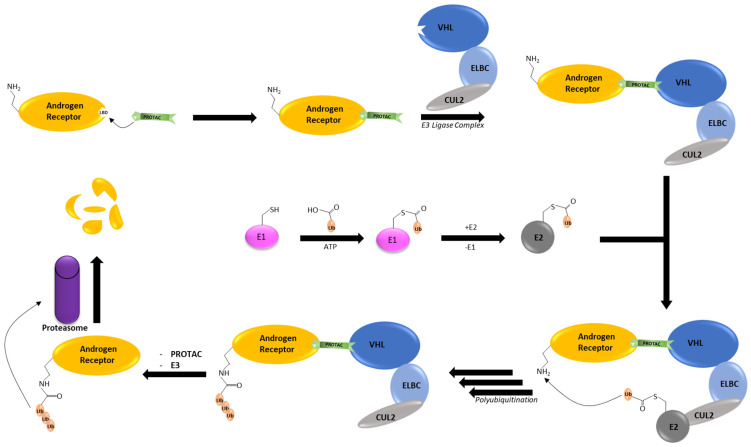
Mechanism of action behind AR degradation by PROTACs by hijacking the ubiquitin–proteasome system. The PROTAC forces a ternary complex between the AR and the E3 ligase; von Hippel–Lindau (VHL) E3 ligase is used as an E3 ligase example. E1 activates ubiquitin through the adenylation of ubiquitin’s C-terminus, resulting in the linkage via a thioester bond to the catalytic cysteine residue on the E1 enzyme. Subsequently, ubiquitin is transferred from E1 to the ubiquitin-conjugating enzyme (E2) through transthioesterification. E2 then binds to VHL’s E2-ubiquitin-binding domain for the transfer of ubiquitin to the *N*-terminal of the AR via the magic thioester exchange reaction.

As mentioned in Section 1.3, degrading the AR has recently gained interest in the search for treating castration-resistant prostate cancer. Furthermore, recent reports indicate that castration-resistant prostate cancer cells exhibit high AR protein stability, thus compromising the efficacy of AR antagonists [34]. This form of drug resistance is likely to be overcome by AR degraders. The most clinically advanced strategy for degrading the AR is the use of heterobifunctional PROTACs to bring the AR close to an E3 ligase for ubiquitination. The AR was chosen for PROTAC investigation from the outset due to its well-characterized association with androgens and its role in the development and progression of prostate cancer [35]. Multiple PROTACs have demonstrated this, despite their unique differences in mechanisms. Figure 2 illustrates the general mechanism of AR degradation catalyzed by PROTACs by hijacking the ubiquitin-proteasome system. Specifically, AR degradation begins when a PROTAC promotes the formation of a ternary complex between the E3 ligase and the AR. Once the ternary complex is formed, a chain of ubiquitin molecules is gradually transferred to the *N*-terminal of the AR, marking it for degradation by the 26*S* proteasome.

Several AR PROTACs have been identified. Their antiproliferative potency (IC_50_ values) against AR-positive prostate cancer cells, along with the degradability of representative androgen receptor PROTACs, have been summarized [36]. Two AR PROTACs have advanced to phase II clinical trials, and other four have entered phase I clinical trials since 2020. During this period, a significant focus among scientists was on developing orally active PROTACs by utilizing CRBN binders in combination with shorter and rigid linkers. The advances of AR PROTACs in clinical studies, AR binders, E3 ligase binders, and linkers since 2020 are overviewed below.

### 3.1. Advances in Clinical Studies

As of now, only ARV-110 and ARV-766 have advanced to phase II clinical trials as AR PROTACs. Additionally, four other AR PROTACs (three for metastatic castration-resistant prostate cancer, one for androgenic alopecia and acne) have entered phase I clinical trials [22,37]. Given the substantial interest from both academia and industry, it is anticipated that more AR PROTACs will undergo clinical studies. These promising results stem from meticulous empirical structure–activity relationship investigations. Hence, having crystal structures of AR–PROTAC–Ligase ternary complexes is highly desirable for providing rational guidance in future PROTAC design [37].

#### 3.1.1. ARV-110 (Bavdegalutamide)

**Figure 3:**
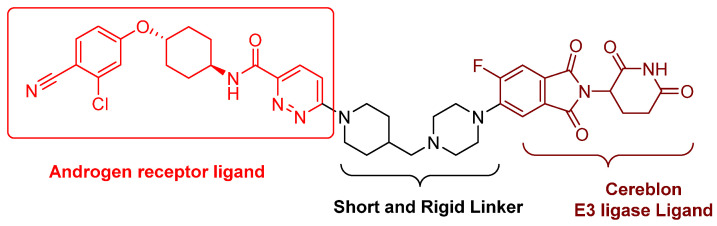
Chemical structure of ARV-110.

ARV-110 (bavdegalutamide), developed by Arvinas, stands as one of the most pioneering PROTACs to enter clinic trials in 2019 and is currently the most advanced PROTAC in phase II clinical trials for castration-resistant prostate cancer. Its chemical structure (Figure 3) was disclosed on 11 April 2021 at the annual American Association for Cancer Research meeting. ARV-110 is an orally active AR PROTAC, characterized by an aryloxy cyclohexane AR antagonist, a short and rigid piperidine–piperazine linker, and a cereblon E3 ligase ligand (Figure 3). According to Ian Taylor, Avinas’s Chief Scientific Officer, this clinically advanced AR PROTAC features a shorter and more rigid linker compared with those targeting other proteins and early academic versions. The phase I trial (NCT03888612) results suggested that ARV-110 is well-tolerated by the participants at doses up to 420 mg, effectively degrading AR in prostate tumors and suppressing tumor growth. Consequently, its phase II clinical study (ARDENT) commenced in October 2020 at a dose of 420 mg [38]. The phase II clinical results were presented at the 2022 American Society of Clinical Oncology Genitourinary Cancer Symposium. ARV-110 consistently demonstrates its antitumor efficacy among prostate cancer patients with T878X and H875Y mutations [39]. Approximately 50% of patients with these mutations treated with ARV-110 experienced a significant reduction in prostate-specific antigen levels, an unexpected result since ARV-110 was not initially designed to degrade mutated ARs.

Additionally, a phase I clinical trial (NCT05177042) commenced on 1 February 2022 to evaluate the safety, tolerability, and pharmacokinetics of the combination of ARV-110 plus abiraterone in participants with metastatic prostate cancer. As of now, no results from this trial have been disclosed.

#### 3.1.2. CC-94676 (AR-LDD, NCT04428788)

The chemical structure of CC-94676 has not been disclosed. Initially developed by Celgene, Inc. and later by Bristol Myers Squibb, it entered phase I clinical trials on 22 June 2020, with the objective of evaluating safety, tolerability, pharmacokinetics, and pharmacodynamics in patients with metastatic castration-resistant prostate cancer. As of now, no study results have been published.

#### 3.1.3. ARV-766 (NCT05067140)

**Figure 4:**
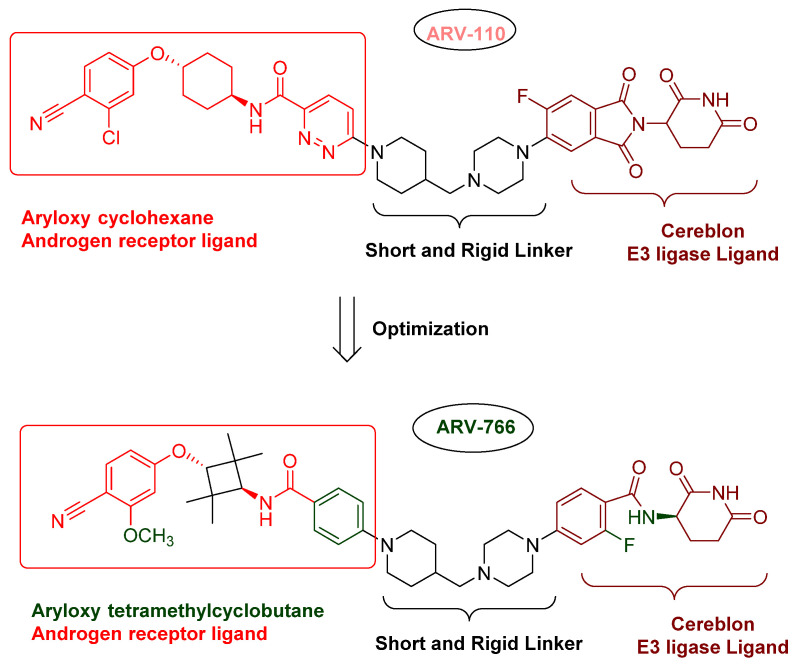
Chemical structure comparison between ARV-110 and ARV-766.

The chemical structure of ARV-766 (Luxdegalutamide), sponsored by Arvinas Inc., was disclosed at the American Association for Cancer Research (AACR) Annual Meeting on 14–19 April 2023 (Poster #AACR23). Enantiopure ARV-766, designed for optimal genotype coverage, was developed by optimizing the AR ligand and E3 ligase ligand of ARV-110 (Figure 4). Preclinical experimental data demonstrated that ARV-766 robustly suppresses tumor growth even under high concentrations of androgen. Clinical studies of ARV-766, as a monotherapy or in combination with abiraterone, have recently progressed from phase I to phase II in patients with metastatic castration-resistant prostate cancer.

#### 3.1.4. HP-518 (NCT05252364)

The chemical structure of HP-518 has not been disclosed. As an orally bioavailable PROTAC, it entered a phase I clinical trial in 2022 in Australia for patients with metastatic castration-resistant prostate cancer. Hinova Pharmaceuticals, Inc. is the sponsoring company.

#### 3.1.5. AC176 (AC0176) (NCT05241613)

AC176-001 is another orally active AR degrader. AC176-001 (the chemical structure has not been disclosed yet) entered a phase I clinical trial in 2022 for participants with metastatic castration-resistant prostate cancer who have received at least two prior treatments (NCT05241613). This clinical study aims to assess safety, tolerability, pharmacokinetic, pharmacodynamic, and preliminary anti-tumor efficacy in patients. The sponsoring company is Accutar Biotechnology, Inc.

#### 3.1.6. GT-20029 (NCT05428449)

The chemical structure of GT-20029 is undisclosed. Sponsored by Suzhou Kintor Pharmaceutical, Inc., it entered a phase I clinical trial in 2022 for the potential treatment of acne vulgaris and androgenetic alopecia.

### 3.2. Advances in AR Binders

**Figure 5:**
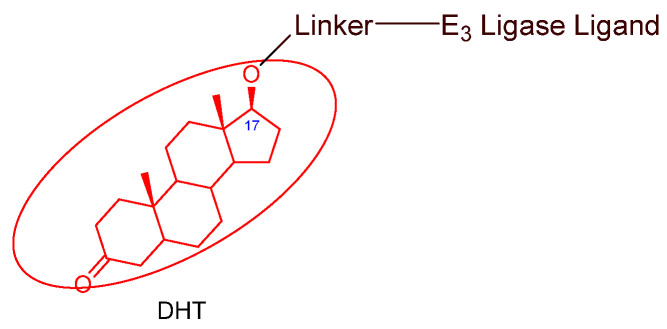
General structure of DHT-based AR PROTACs.

**Figure 6:**
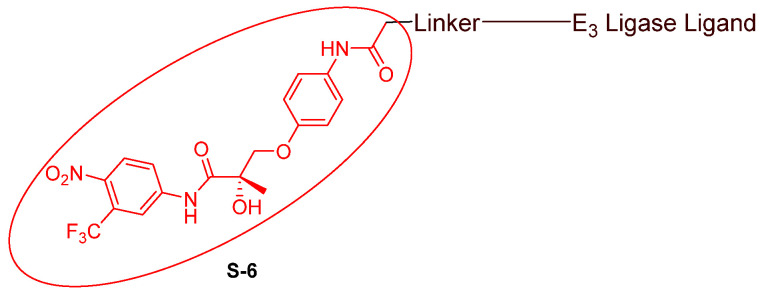
General structure of S-6-based AR PROTACs.

**Figure 7:**
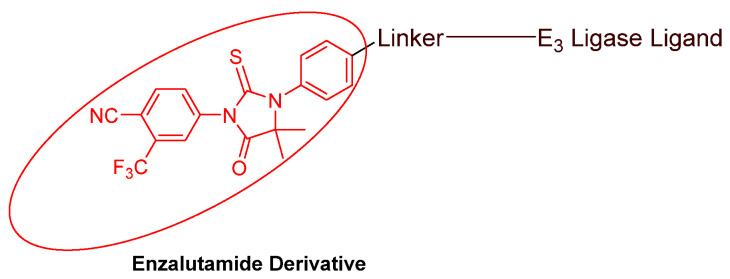
General structure of enzalutamide-based AR PROTACs.

**Figure 8:**
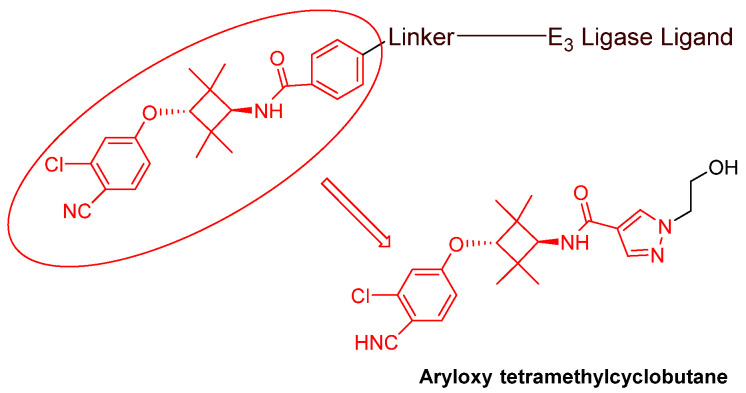
General structure of aryloxy tetramethylcyclobutane-based AR PROTACs.

**Figure 9:**
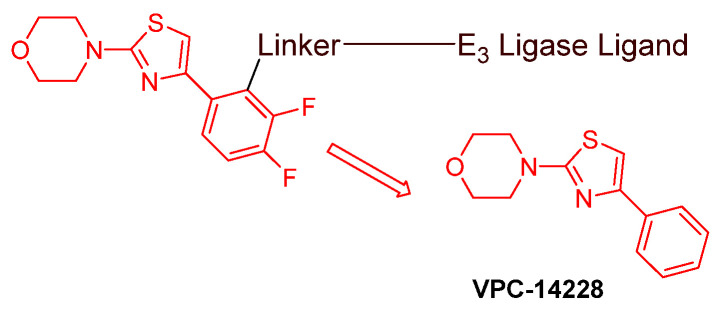
General structure of VPC-14228-based AR PROTACs.

**Figure 10:**
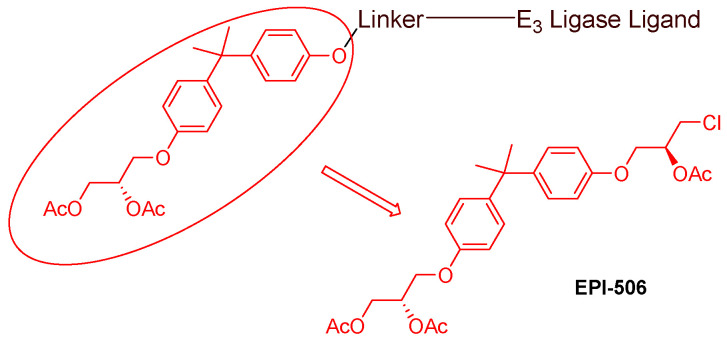
General structure of EPI-506-based AR PROTACs.

Even though numerous AR PROTACs have been investigated both preclinically and clinically, only a few AR agonists and antagonists have been used in the reported PROTACs. The following groups of compounds have been used as AR ligands in PROTACs:

- Endogenous androgen as the ligand binding domain binder: dihydrotestosterone. This AR agonist exhibits very high binding affinity to the ligand binding domain, and its hydroxyl group at C-17 makes it easy to introduce various linkers via an ester bond (Figure 5) [40,41,42].
- Nonsteroidal AR agonists: S-6 and derivatives. This group of agonists exhibits greater anabolic activity than androgenic activity. Figure 6 illustrates that the linker is easily incorporated into the *N*-acetyl group [43].
- Nonsteroidal AR antagonists binding to the ligand binding domain: enzalutamide (Figure 7) and aryloxy tetramethylcyclobutane (Figure 8). Enzalutamide is a currently marketed AR antagonist for castration-resistant prostate cancer. Enzalutamide-based PROTACs, such as ARCC-4, exemplify this class [21]. Aryloxy tetramethylcyclobutane was identified by Pfizer as a highly potent AR antagonist using cell-based high-throughput screening [44]. In addition to serving as the AR recruiter for ARC-766 developed by Arvinas, it has been successfully incorporated into a group of AR PROTACs, such as ARD-69, by Dr. Wang’s research group [45]. Aryloxy cyclohexane (Figure 3) and its amine derivative were used as AR binders in ARV-110 and ARD-2585 [46], which are two orally active PROTACs with exceptional potency. Technically, mutants and variants lacking the LBD render this group of AR degraders ineffective. However, treatment with ARV-110 led to about a 50% decrease in prostate-specific antigen in the treated patients with two mutations on the LBD.
- AR DBD binders: VPC-14228. This group of AR ligands has less specificity for the AR due to the highly conserved DBD structure found in all nuclear receptors. MTX-23 is a reported AR PROTAC with VPC-14228 as an AR ligand that binds to DBD, enabling the degradation of ARV7 (Figure 9) [47]. The surface-exposed region on the AR DBD has been identified as an alternative druggable pocket for potential AR antagonists. VPC-14228 binds to this pocket through hydrogen bonding between its morpholine group and Tyr594, hydrophobic interactions between the thiazole ring and Val582/Phe583, and hydrophobic interactions between the phenyl ring and the aliphatic side chains of Arg609 and Lys610 [47].
- AR antagonists binding to the *N*-terminal domain: The intrinsic disorder of the *N*-terminal domain poses a challenge to the rational design of *N*-terminal domain binders. The well-established AR *N*-terminal antagonist EPI-506 has been successfully used to create an AR PROTAC BWA-522 and derivatives (Figure 10) [48]. EPI-506 functions as a prodrug of EPI-002. Non-covalent interactions between EPI-002 and the transactivation unit 5 (Tau-5) domain of the *N*-terminal domain have been identified using NMR spectroscopy. This interaction involves a subset of three partially helical regions, namely, the R1 region (residues S341–G371), R2 region (residues L391–G414), and R3 (residues S426–G446) [49]. All-atom molecular dynamics computer simulations further elucidate that EPI-002 binds specifically to the R2 and R3 regions of Tau-5. Notably, amino acid residues W397 and W433 play the most significant role in mediating the interaction between EPI-002 and the R2 and R3 regions of Tau-5 [50].

Since 2020, the spotlight has been on specific AR ligands in the development of AR PROTACs. The primary focuses are (i) Nonsteroidal AR antagonists binding to the ligand binding domain. Notably, aryloxy cyclohexane (depicted in Figure 3) and its amine derivative, featured in ARV-110 and ARD-2585 [46], have emerged as key components in the synthesis of AR PROTACs post-2020. (ii)AR antagonists binding to the DNA binding domain (Figure 9). (iii) AR antagonists binding to the *N*-terminal domain (Figure 10). Developing novel PROTACs with an *N*-terminal or DNA-binding domain AR binder has been proposed to serve as a promising strategy to fight resistance to the current FDA-approved AR antagonists.

### 3.3. Advandes in E3 Ligase Binders

Even though over 600 E3 ligases have been predicted, only a few of them possess fully characterized functions [51]. Four groups of E3 ligases that have most frequently been hijacked by PROTACs to induce protein degradation are von Hippel–Lindau (VHL), cereblon (CRBN), MDM2, and IAP [52]. It has been demonstrated that hijacking the ubiquitin–proteosome system via different E3 ligases provided a significantly different degree of protein degradation, even with an identical AR ligand and linker [33].

#### 3.3.1. MDM2 and IAP Binders

So far, the only MDM2-based AR PROTAC was reported by the Crews research group at Yale University back in 2008 [43]. This early PROTAC uses MDM2 antagonist nutlin as an E3 ligase ligand to recruit MDM2 E3 ligase, which degrades the AR at 10 µM in HeLa cells transiently expressing the AR. In addition to the low potency of this MDM2-based PROTAC, interpreting nutlin PROTAC activity is challenging when nutlin is used as the E3 recruiting portion of the chimera. This is because AR is also a direct substrate of MDM2, and nutlin itself is known to induce the ubiquitination of AR in cancer cells [26]. Therefore, no other MDM2-based AR PROTAC has been reported since then.

Inhibitors of apoptosis protein (IAP) have been used as E3 ligase binders in three AR PROTACs [53,54]. IAP-based PROTACs are also known as IAP-based protein erasers (SNIPERs). SNIPER-13 [53] and SNIPER(AR)-51 [54] have displayed AR degradation activity at micromolar concentrations. Since this group of PROTACs, using IAP inhibitors as warheads to recruit IAP protein, does not demonstrate sufficient potency, IAP-based AR PROTACs have not been further pursued since 2020.

#### 3.3.2. VHL Binders

The recruitment of von Hippel–Lindau (VHL) for AR degradation was the most explored approach during the period of 2018–2021, culminating in several promising AR PROTACs with a VHL ligand warhead. ARCC-4 [21], ARD-69 [45], ARD-61 [55], ARD-266 [56], and A031 [57] are excellent representatives. However, none of them has advanced to clinical studies, and significantly fewer VHL-based PROTACs have been reported since 2022. This is mainly because the VHL ligands in the reported AR PROTACs are peptidomimetics with a molecular weight greater than 400, posing poor physiochemical and pharmacokinetic properties [55].

#### 3.3.3. CRBN Binders

The mechanism of thalidomide teratogenicity was unveiled when it was identified as a ligand for the E3 ligase cereblon (CRBN) in 2010 [58]. Thalidomide and its analogs (e.g., phthalimides or IMiDs) have been used in PROTACs to induce the degradation of ARs, as exemplified by two of the most clinically advanced AR degraders (ARV-110 and ARV-766). Encouraged by the promising clinical data for ARV-110 and the disclosure of the chemical structures of ARV-110 and ARV-766, CRBN binders emerged as the most favorable E3 ligase binders in AR PROTACs since 2020.

Commonly used CRBN ligands include thalidomide and TD-106. These E3 ligase recruiters are superior to VHL E3 ligase ligands due to their smaller molecular weight (~250) and more drug-like properties. So far, only CRBN-based AR PROTACs demonstrate satisfactory oral bioavailability. The meta and para positions in the phenyl ring of these ligands have been used as linker attaching points. In addition to ARV-110 and ARV-766, the representative PROTACs with a CRBN recruiter that have been reported since 2020 include TD-802 [59], PAP508 [60], ARD-2128 [61], ARD-2585 [46], ARD-2051 [62], BWA-522 [48], enzalutamide-based PROTAC [63], and S-6-Based PROTAC [64]. However, PROTACs with CRBN binders lack sufficient selectivity and degrade off-target similar to G1 to S phase transition protein 1 [52,65].

#### 3.3.4. Emerging E2 Ligase Binders

One recent advancement involves recruiting E2 ubiquitin-conjugating enzymes, a core component of the ubiquitin–proteasome system machinery, using an appropriate ligand in PROTACs for AR degradation [66]. It is highlighted that covalent chemoproteomic approaches can be used to quickly identify allosteric covalent ligands as ligase binders in PROTACs.

### 3.4. Advances in Linkers

The length and composition of a PROTAC influence not only its pharmacokinetic but also its pharmacodynamic properties. However, the current consensus is that the linker length and composition must be optimized individually for each PROTAC [67]. This adds to the complexity of PROTAC design, as it is challenging to determine which combination of target protein, linker, and E3 ligase will provide optimal PROTAC degradation. Although there is no agreed-upon strategy for the design of PROTAC linkers, alkyl or PEG (polyethylene glycol) chain linkers are commonly used in PROTACs [67]. Historically, around 65% of PROTACs consist of either a PEG or an alkyl chain linker [33,67]. Other motifs represented in PROTAC linkers include triazoles, piperidines, and piperazines [67]. The prevalence of PEG and alkyl linkers is attributed to their flexibility, easier synthesis, and the ability to easily manipulate the length to optimize PROTAC behavior. In determining the minimum linear length for PROTACs targeting TBKI developed by ARVINAS, alkyl linkers from 7 to 29 atoms were tested, and it was found that degradation was not observed when the linker length was below 12 atoms. Degradation was observed for linkers ranging from 12 to 29 atoms. Burkart and colleagues also noticed that there was a weak correlation between PROTAC linker length and PROTAC efficacy when they compiled data on multiple PROTACs with linear linkers [68]. Specifically, as linker lengths became shorter, protein degradation sharply decreased, probably because of steric hindrance between the target protein and E3 ligase complex, making the ternary complex impossible. On the other hand, degradation gradually decreases as linkers become longer, likely because the E3 ligase would have a harder time ubiquitinating the protein. No specific conclusion about the optimal length was provided. However, with two different PROTACs, one with a 9-atom linker and the other with a 16-atom linker, it was observed that both were equally potent.

Exploring AR PROTACs reported since 2020 reveals that potent representatives, such as ARD-61 [55], TD-802 [59], ARD-2128 [61], ARD-2585 [46], BWA-522 [48], and ARV-766, feature rigid heterocyclic linkers ranging from 6 to 12 atoms in length. Most of them use piperidine and piperazine as rigid linkers. While ARV-110 was initially speculated to have a long flexible linker, the actual linker is a short and rigid combination of piperidine and piperazine. These rigid linkers endorse not only the degradability of PROTACs, as evidenced by the reported DC_50_ and D_max_ values, but also the kinetic profile [46].

## 4. Hydrophobic Tagged Chimeric AR Degraders

Similar to PROTACs, hydrophobic tagged degraders can decompose AR, albeit through a different degrading mechanism. The hydrophobic tagged AR degraders have been designed and synthesized by connecting a hydrophobic adamantyl group to the AR agonist RU59063 or its derivatives through a flexible linker [69,70]. Mechanistically, the bulky hydrophobic adamantyl group adheres to the surface of the AR, resembling a partially denatured hydrophobic region of the AR. This adherence triggers AR degradation through ubiquitin proteolysis, facilitated by the HSP70/CHIP complex. However, the hydrophobicity of the tag is directly associated with low oral bioavailability. As of now, no hydrophobic tagged chimeric protein degraders have entered clinical studies [71]. Additionally, no new hydrophobic tagged AR degraders have been reported since 2021.

## 5. Monomeric AR Degraders with Diverse Mechanisms

In addition to PROTACs and hydrophobic tagged AR degraders, numerous monomeric AR degraders, characterized by relatively low molecular weight, have been demonstrated to reduce the expression of the AR and its variants. Monomeric AR degraders are smaller, making it easier to ensure favorable pharmacokinetic properties. They have garnered more attention from medicinal chemists since 2020. However, there are no rational guidelines available for designing and screening monomeric AR degraders.

### 5.1. UT Series

**Figure 11:**
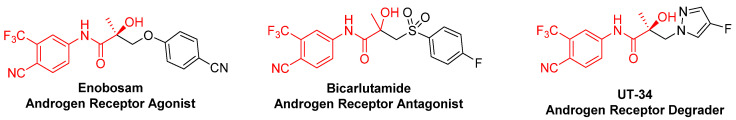
Chemical structures of enobosam, bicarlutamide, and UT-34. The red color indicates the same chemical moiety shared by the three compounds.

The most investigated group of monomeric AR degraders is the UT series, also known as selective AR degraders (SARD) [35,72,73,74,75]. This selective and orally active degrader group was developed through chemical manipulation of AR antagonists (e.g., bicarlutamide) and agonists (e.g., enobosam) (Figure 11), which bind to the ligand binding domain. Surprisingly, some UT series compounds (e.g., UT-34) bind to both the ligand binding domain and the *N*-terminal domain of AR. Upon binding to the AF1 region of the *N*-terminal domain of both the full-length and ligand binding domain-truncated AR, the UT series promotes the degradation of both the full-length AR and the ligand binding domain truncated variant (AR-V7) via the proteasomal pathway. The UT series has been evidenced by in vitro and in vivo experimental data to be more potent than enzalutamide in enzalutamide-resistant castration-resistant prostate cancer cell models and xenografts, especially in ligand binding domain-truncated AR-V7 xenograft [72,73,74,75]. UT-34 (ONCT-534) has advanced into phase I/II clinical studies (NCT05917470). Further improvement of their pharmacokinetic properties in vivo is the direction for moving the UT series toward clinical use. Very recently, UT-143 has been reported to irreversibly suppress both full-length AR and AR-V7 transactivation, with an IC_50_ value of 150 nM. This suppression occurs through the selective and covalent binding to two cysteines (C406 and C327) via Michael additions in the AF-1 region [76]. The mechanism underlying the deactivation of both full-length AR and AR-V7 by UT-143 is associated with disrupting the formation of liquid–liquid phase separation condensates and inducing subsequent conformational change.

### 5.2. AR Degraders via the Dissociation of Chaperone Protein

The induction of the dissociation of chaperone protein HSP90 from the AR can promote AR degradation, as chaperone protein HSP90 is indispensable for stabilizing the AR. Some reported AR degraders achieved through the dissociation of the chaperone protein are summarized below.

#### 5.2.1. ASC-J9 (NCT01289574)

**Figure 12:**
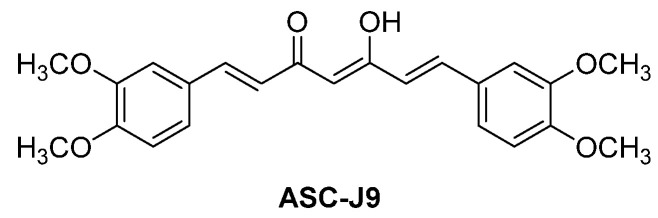
Chemical structure of ASC-J9.

ASC-J9 (Figure 12), an AR degrader developed by AndroScience, has entered phase II clinical trials for potential clinical use for men with androgenetic alopecia. Initially identified as a potential AR antagonist [77], it was later established as an AR degrader [78]. Specifically, the conformational change induced by the binding of ASC-J9 to the AR leads to the dissociation of the chaperone protein HSP90. Consequently, the unprotected AR undergoes ubiquitination for degradation via proteasome. The follow-up investigations suggest that the dissociation of AR induced by ASC-J9 from coregulators like ARA55 or ARA70 promotes the association between AR and MDM2 E3 ubiquitin ligase, ultimately leading to AR degradation via the ubiquitin–proteasome system [79,80].

#### 5.2.2. Niclosamide and ARVibs

Niclosamide, an antihelminthic drug, was identified as an AR-V7 degrader after screening 1120 FDA-approved marketed drugs [81]. Its derivatives, ARVibs [82], exhibit improved pharmacokinetic properties and can degrade both the full-length AR and AR-V7 protein. The mechanism behind AR/AR-V7 degradation involves the ubiquitin–proteasome pathway. Specifically, ARVibs suppress the expression of chaperone protein HSP70 and promote the translocation of STUB1 E3 ligase into cell nuclear. This results in the binding of AR/AR-V7 to the STUB1 E3 ligase, followed by ubiquitination and degradation of AR/AR-V7.

#### 5.2.3. Geldanamycin Analogs

17-Allylamino-17-demethoxygeldanamycin (17-AAG), a derivative of the naturally occurring geldanamycin, has been proven to inhibit HSP90 chaperone function and degrade both the wild-type and mutant ARs, which requires HSP90 for folding. In an in vivo experiment using a CWRSA6 prostate cancer xenograft, the administration of 17-AAG at 50 mg/kg reduced AR expression by 80% [83]. The potency and toxicity of 17-AAG were assessed in patients with metastatic castration-resistant prostate cancer in a two-stage phase II trial [84]. It was concluded that 17-AAG did not show any potential regarding prostate-specific antigen response at the end of the first stage, leading to the termination of further enrollment.

17-DMCHAG (17-(6-(3,4-dimethoxycinnamamido)hexylamino)-17-demethoxy-geldanamycin), a newer analog of geldanamycin, has been demonstrated to dissociate the HSP90 chaperone protein from the AR, leading to AR degradation via the ubiquitin–proteasome system [85]. No further research on geldanamycin analogs as AR degraders has been reported since 2020.

### 5.3. Other Monomeric AR Degraders

Several synthetic and naturally occurring compounds have been identified using Western blotting experiments to have the ability to reduce AR levels via the ubiquitin–proteasome system. However, many of them lack a clear degradation mechanism [22]. Two examples are shown here. A darolutamide derivative has been found to suppress the activity of both wild-type AR and F876L mutants. It effectively downregulates the expression of full-length AR and ligand-binding domain-truncated AR-V7, exhibiting superior anti-tumor efficacy compared with enzalutamide against castration-resistant VCaP xenografts [86]. Galeterone and its derivatives have a unique capability to suppress CYP17, antagonize AR, and induce AR degradation. They selectively degrade AR by interfering with the balance between AR ubiquitination and deubiquitination in the ubiquitin–proteasome system [87]. The phase III clinical trial of galeterone indicated insufficient efficacy toward AR-V7-positive metastatic castration-resistant prostate cancer. However, modifications at the C3 and C17 positions have led to more promising derivatives. Additionally, some molecules have demonstrated a dual mechanism of action by suppressing the androgen/AR signal pathway and promoting AR degradation.

## 6. Molecular Glues to Degrade AR

Molecular glues (also known as proximity inducers), which were originally discovered serendipitously, have rapidly emerged as an innovative strategy for targeted protein degradation since 2020 [88,89]. To target the AR for degradation, molecule glues induce proximity between the AR and ubiquitin ligases, forming the ternary complex of the molecular glue, the AR, and ubiquitin ligases. Through this process, molecular glues bring the AR for degradation via the ubiquitin–proteasome system. VNPP433-3*β*, a new-generation galeterone analog, is a molecular glue that facilitates physical proximity between the AR and MDM2 E3 ligase, resulting in AR ubiquitination and degradation by proteasome [90]. Generally, molecular glues have a low molecular weight (less than 500 Da), likely possessing favorable pharmacokinetic properties according to Lipinski’s rule of five. However, there are currently no general drug discovery strategies and systematic evaluations available for molecular glue AR degraders. VNPP433-3*β* is the only reported molecular glue capable of degrading the AR up to this point.

## 7. Autophagic Degradation of AR

It has been reported that the ubiquitination of target proteins can lead to either proteasome degradation or autophagic degradation [91]. Ubiquitin codes, created by different ubiquitin molecules, signal distinct degradation pathways for target proteins. For example, K48 ubiquitin chains mainly flag proteins for proteasomal degradation, while K63 ubiquitin chains promote lysosomal degradation [92,93]. Targeting proteins for autophagic degradation can be achieved by directing the ubiquitinated protein to autophagosomes through the autophagy receptor p62, followed by degradation within lysosomes. Riluzole, a marketed drug for the treatment of amyotrophic lateral sclerosis, has been reported to degrade full-length ARs, mutant ARs, and AR-V7 [94]. Its degrading mechanism involves (i) promoting selective autophagy via boosting the interaction between the AR and autophagy receptor p62 and (ii) activating IRE1*α* and ATF6*α* endoplasmic reticulum stress signaling arms. This represents an emerging strategy for degrading ARs, and riluzole is the only example found so far.

## 8. Conclusions and Future Perspective

The AR remains a crucial target for castration-resistant prostate cancer. While various AR antagonists have been approved by the U.S. FDA and demonstrated survival benefits for patients at different stages of prostate cancer, the persistent challenge lies in drug resistance. To address this limitation, there is a growing body of evidence supporting AR degradation as a viable strategy. Various approaches, including PROTACs, hydrophobic tags, monomeric degraders, molecular glues, and autophagic degraders, have been explored for this purpose. As summarized in Table 1, several AR degraders have reached clinical trials. Notably, two AR PROTACs have advanced to phase II clinical trials, while four others are in phase I clinical trials, underscoring the increasing interest since 2020. A focused effort has been directed toward developing orally active PROTACs, using CRBN binders and shorter, more rigid linkers. Novel PROTACs with *N*-terminal or DBD AR binders have emerged as a promising strategy to combat resistance to currently FDA-approved AR antagonists. The development of these promising degraders stems from meticulous empirical structure–activity relationship studies. However, the crystal structures of AR–PROTAC–ligase ternary complexes will be very critical to provide essential guidance for future PROTAC design. Additionally, the incorporation of artificial intelligence, including machine learning and deep learning algorithms, is recommended to expedite the discovery of effective and drug-like AR degraders [95].

Innovative strategies, such as PROTAC prodrugs that have been proposed to mitigate on-protein target off-tumor toxicity [96], PROTAC prodrugs demonstrating activation by radiotherapy within tumors [97], advances in PROTAC delivery systems [98], the expansion of ubiquitin E3 ligases for PROTACs [99], and a deeper understanding of ternary complex structures should be recognized as critical areas for further exploration of AR degraders.

## Figures and Tables

**Table 1:** AR degraders that have advanced to clinical studies.

Degrader	Other Name	Treatment	Phase	Sponsor	ClinicalTrials.gov Identifier
ARV-110	bavdegalutamide	Prostate cancer	I and II	Arvinas Inc.	NCT03888612
ARV-766	luxdegalutamide	Prostate cancer	I and II	Arvinas Inc.	NCT05067140
CC-94676	AR-LDD	Prostate cancer	I	Bristol Myers Squibb	NCT04428788
HP-518	–	Prostate cancer	I	Hinova Pharmaceuticals Inc.	NCT05252364
AC176	AC0176	Prostate cancer	I	Accutar Biotechnology Inc.	NCT05241613
GT-20029	–	Acne vulgaris and androgenetic alopecia	I	Suzhou Kintor Pharmaceutical, Inc.	NCT05428449
UT-34	ONCT-534 GTx-534	Prostate cancer	I & II	Oncternal Therapeutics, Inc.	NCT05917470
ASC-J9	–	Acne vulgaris	II	AndroScience Corp.	NCT00525499
17-AAG	–	Prostate cancer	II	National Cancer Institute	NCT00118092
Galeterone	–	Prostate Cancer with AR-V7	III	LTN Pharmaceuticals, Inc.	NCT02438007

## Data Availability

Not applicable.
